# Supplementary material for: On the preservation of vessel bifurcations during flow-mediated angiogenic remodelling
Source: PLoS Comput Biol. 2021 Feb 4;17(2):e1007715. doi: 10.1371/journal.pcbi.1007715 (PMC7909651; doi:10.1371/journal.pcbi.1007715)
Supplement: S1 Text — (DOCX) [file pcbi.1007715.s014.docx]

**S1 Text. Stability is robust but does vary across changes in boundary condition, network geometry, and shear stress difference**

The final task of our study was to demonstrate that the stability behaviour described previously is not exclusive to our pressure-driven A branch model with periodic cell conditions. Hence, we performed similar stability analysis across different boundary conditions and vessel network geometries. The first variation of the A branch model included a prescribed flow condition at the inlet as opposed to prescribed pressure (S10 File). In this model, the pressure at the inlet varies in order to set a prescribed flow at the inlet, which was set to the same initial incoming flow in the pressure-driven model. Prescribing inlet flow instead of pressure had no effect on the stability at the flow-convergent bifurcation, as bifurcation loss vs. *α* over time was identical to the pressure-driven results (S11 Fig). Next, we sought to determine the impact on the EC boundary conditions on stability by holding the number of incoming cells constant, meaning the total number of ECs within the network varied in order to match this condition (as opposed to the periodic condition where the total number of ECs was held constant) (S12 File). Simulations with this condition resulted in a similar asymmetric saddle-shape while sweeping over the range of *α*; however, the global minimum of bifurcation loss was shallower (i.e., less stable) when compared to the periodic cell conditions (S13 Fig). In our final variation of the A branch model, we sought to determine how the initial shear stress difference at the bifurcations impacted stability. In the original A branch model, the shear stress difference at the bifurcation had an initial ratio of 2:1, proximal to distal, due to the distal path being twice the length (and hence twice the resistance) of the proximal path. We therefore created a new version of the A branch model in which the distal path was 10× the length of the proximal path, creating an initial shear stress ratio of 10:1. Stability analysis with this model resulted in a similar asymmetric saddle to the 2:1 case, although the global minimum was shallower (less stable) and shifted slightly to the left centred around *α* = 0.375 (S14 Fig).

The A branch model is a basic representation but still includes some “network effects” as flow is split at the flow-divergent bifurcation before re-joining at the flow-convergent bifurcation. This model has some advantages, specifically in that cell behaviour at the flow-convergent bifurcation doesn’t propagate to the flow inlet as cells in both paths combine at the flow-divergent bifurcation. However, varying the shear stress difference at the bifurcation in this model is not straightforward and requires a change in geometry (i.e., change in path lengths between the two branches). Thus, we created a simplified model of a flow-convergent bifurcation (hence referred to as the Y branch model) in which incoming flow from a left and right branch combine at a single bifurcation before exiting via the outlet (S15 File). Using this model, we can set the inlet pressure in both the left and right branches to be equal in order to achieve a 1:1 shear stress ratio (i.e., no shear stress difference) at the bifurcation (S16 Fig). Stability across the range of a in this model was very similar to the pressure-driven A branch model, with a global minimum across values between 0.3 and 0.6. The branch probabilities in this model initialise at 0.5 as the cell number and shear stress in both branches is the same and exhibit similar competitive oscillations which result in bifurcation stability (S17 Fig). Finally, we implemented the Y branch model with inlet flow conditions in both branches that allows us to vary the initial shear stress difference at the bifurcation at ratios of 1:1, 1:10, and 1:20 (S18 File, S19 File, S20 File). Each of these cases resulted in a similar saddle shape in the stability surface, although this saddle became shallower (less stable) and shifted to the left (towards *α* = 0.0) as the shear stress difference increased (S21 Fig). While there was no difference in mean diameter between the two branches with a shear ratio of 1:1, increasing the ratio between shear stress led to larger differences in diameter between the two branches (S22 Fig).

Our stability findings were robust across changes in boundary condition, vessel geometry, and shear stress initialisation at the bifurcation, strongly suggesting that our findings are truly inherent to physical system we are representing and not a manufactured outcome of any one particular model configuration. Inlet flow conditions made no impact of stability at the flow-convergent bifurcation in the A branch model, most likely due to the face that cells from both branches recombine at the flow-divergent bifurcation rendering the inlet free from EC behaviour at the flow-convergent bifurcation. In the inlet flow version of the Y branch model, which does not include this recombining effect, we found slightly different results but the classic asymmetric saddle shape of stability remained intact. Changing the initial conditions for shear stress (and hence shear stress probability) could affect the nature of the competitive oscillations between the two components; however, when we varied these initial conditions (either by changing the resistance of the distal branch in the A branch model or the flow ratio between branches in the Y branch model) the classic asymmetric saddle shape of stability was maintained albeit more shallow and shifted to the left towards *α* = 0.0. With an increased initial shear stress difference, simulations that favoured junction forces resulted in fewer bifurcations lost, although results were still not as stable when compared to simulations with less of a shear stress imbalance. Changes in the boundary condition governing the number of incoming cells had the greatest impact on bifurcation stability. Simulations which held the amount of incoming cells constant exhibited a general decrease in the total number of cells within the system, and although we found the same asymmetric saddle shape the bifurcation was inherently more unstable with 40% of simulations losing the bifurcation by day 5 of migration (as compared to around 20% found with the Periodic cell condition). These findings suggest that one of the most significant regulators of bifurcation preservation may be the level of new incoming cells: a steady source of new cells greatly enhances the chance of a bifurcation remaining during remodelling. Likewise, a bifurcation which is experiencing reduced or disrupted levels of incoming cells may be more prone to instability and regression. At this point it is unclear what acts as the source of cells in the developing network in vivo. Proliferation may be isolated to the sprouting front, meaning new ECs must arrive via travelling down the network to the remodelling zone. Additionally, large vessels such as veins could also act as a reservoir of new cells. More experimental evidence is required in characterising the source of new ECs in the developing retina in order to investigate the matter further.
