# Supplementary figures and images for: On the preservation of vessel bifurcations during flow-mediated angiogenic remodelling
Source: PLoS Comput Biol. 2021 Feb 4;17(2):e1007715. doi: 10.1371/journal.pcbi.1007715 (PMC7909651; doi:10.1371/journal.pcbi.1007715)

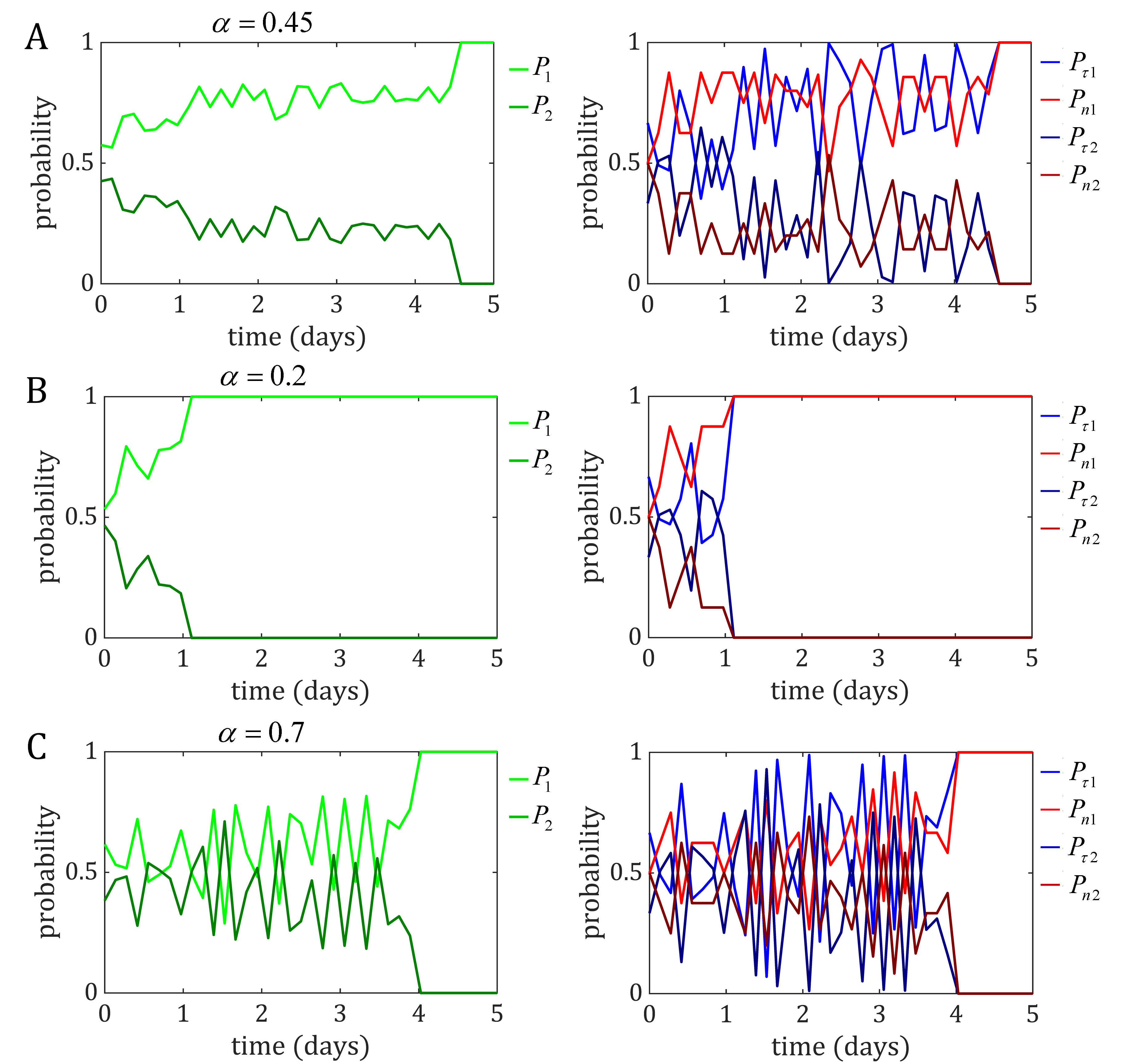

Supplement: S1 Fig — Each result was obtained from the same random seed number with α values of 0.45 (A), 0.2 (B), and 0.7 (C). In all cases, there were temporary oscillations between shear stress and cell number probability while the bifurcation remained stable, but in each case a branch probability reached maximum likelihood (i.e., equal to 1) and the bifurcation was lost. (TIF) [file pcbi.1007715.s015.tif]

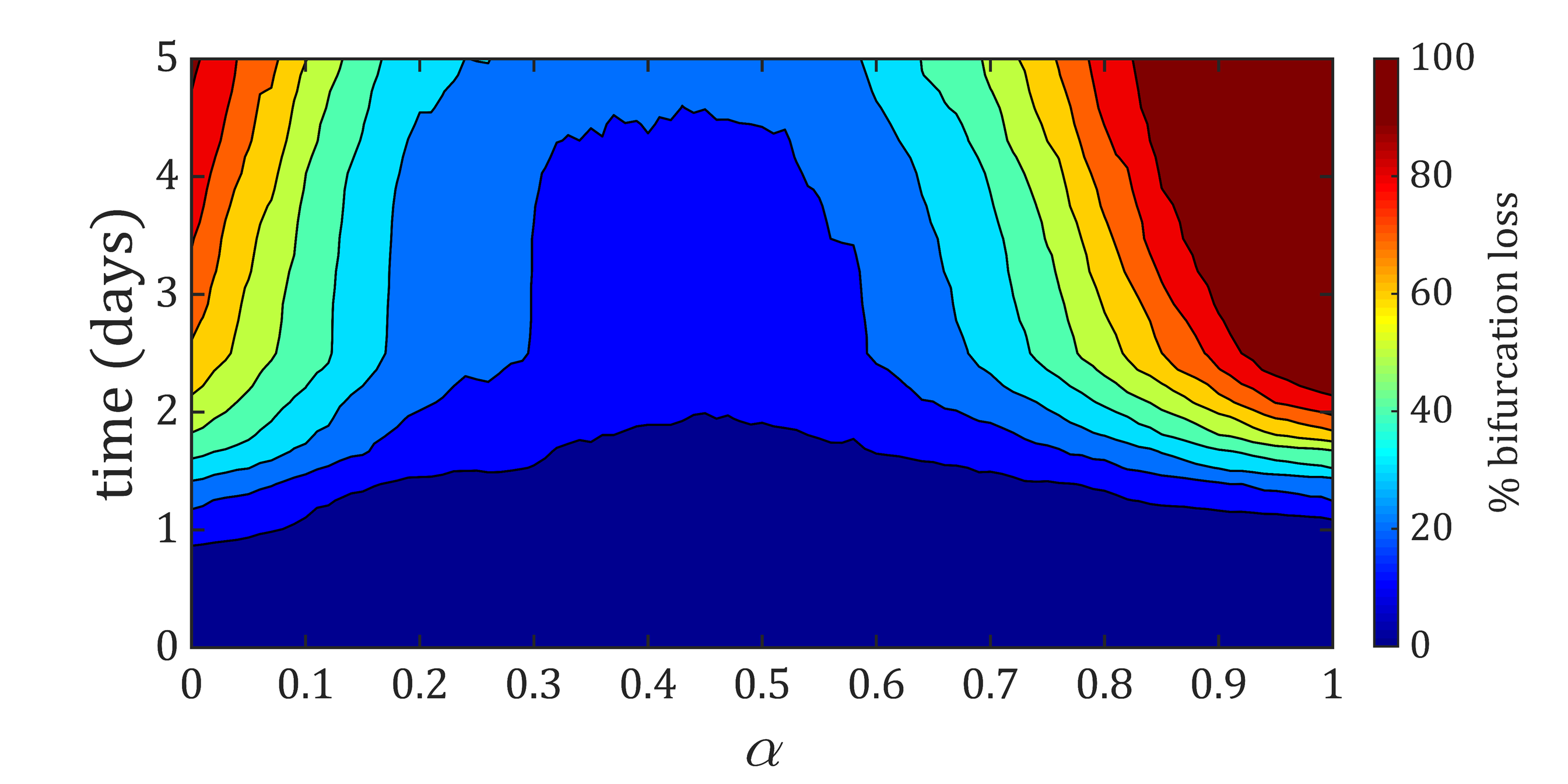

Supplement: S2 Fig — Similar stability analysis as found in Fig 3 with the A branch under inlet flow boundary conditions. Inlet flow was prescribed to match the same initial incoming flow in the pressure-driven formulation. Stability results were identical in the inlet flow version of the model when compared to the pressure-driven formulation. (TIF) [file pcbi.1007715.s016.tif]

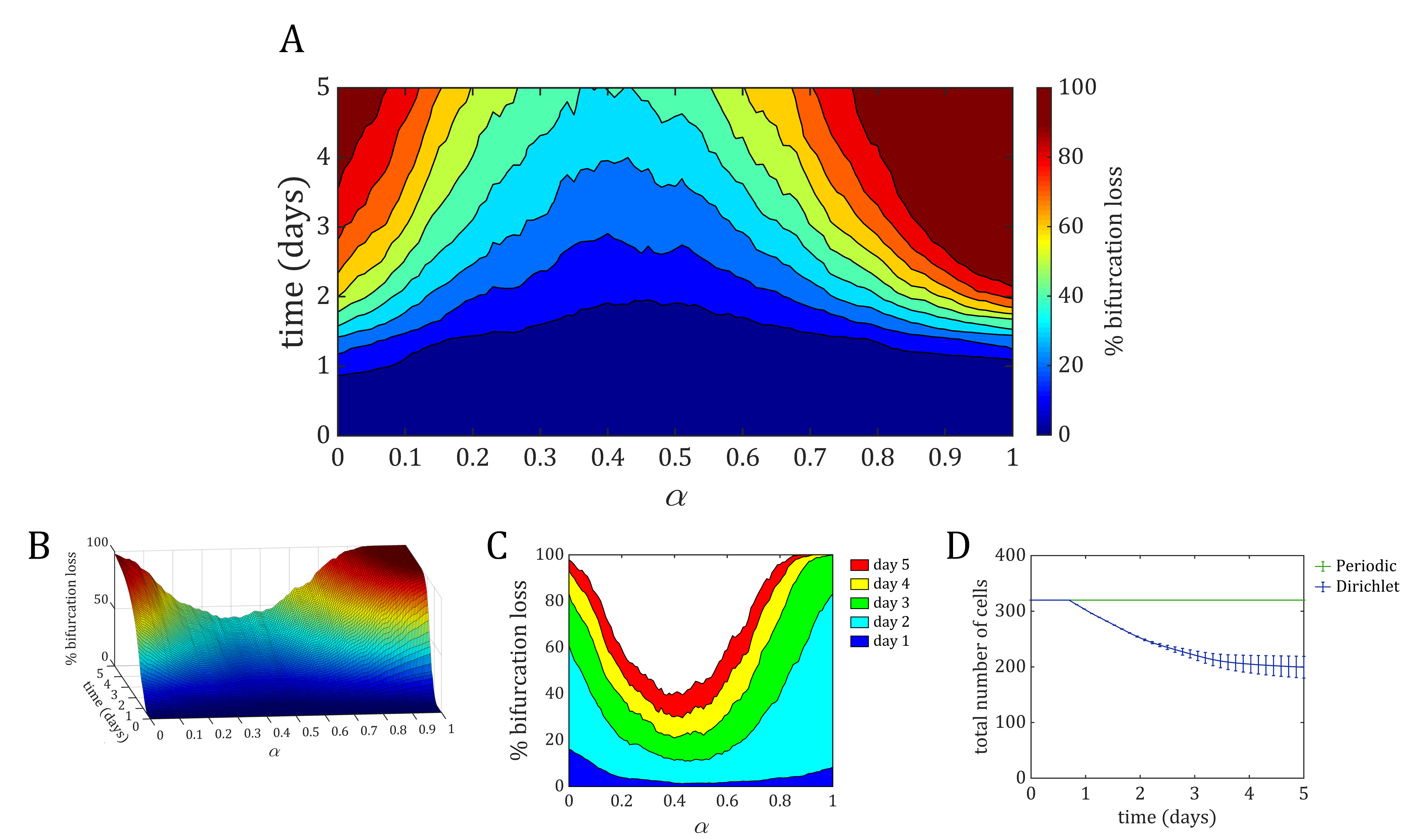

Supplement: S3 Fig — A similar sweep through the values of α to Fig 3 but with Dirichlet cell boundary conditions applied to the model rather than periodic boundary conditions. These boundary conditions enforce that in number of cells incoming to the network (at the flow outlet) was held constant throughout the simulation. This results in the total number of cells within the domain changing over time as this condition is enforced. Simulations using the Dirichlet boundary condition resembled those of the periodic boundary condition but were generally less stable. (A) The contour plot of bifurcation stability vs. α over time shows a similar global minimum of stability around α = 0.4, but even within this stable region more simulations lost the bifurcation at earlier points in time compared to simulations with the periodic boundary condition. (B) The surface formed by bifurcation stability vs. α over time resembled a similar asymmetric saddle shape but was much steeper, especially in the more stable region, indicating that this region was more unstable when compared to the periodic boundary conditions. (C) Similar to the periodic boundary condition case, the majority of bifurcations were lost during day 2 of migration. However, simulations with the Dirichlet condition lost more bifurcations at later days (days 3, 4, 5) when compared to periodic simulations. (D) The total amount of cells in the domain for 1000 simulations at α = 0.45 for both the Periodic and Dirichlet cell condition. The total number of cells remains constant with the Periodic condition while decreasing over time with the Dirichlet condition. (TIF) [file pcbi.1007715.s017.tif]

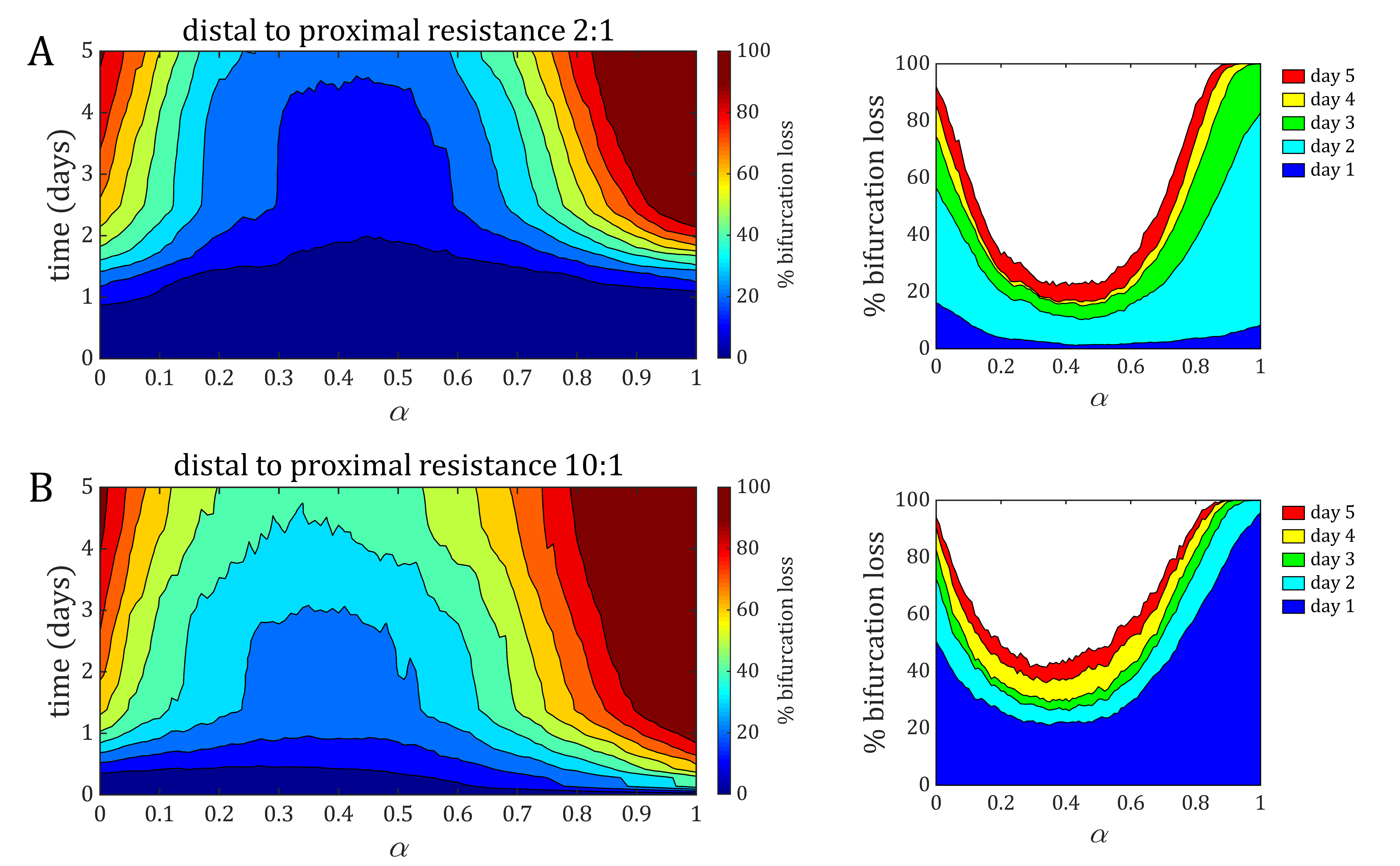

Supplement: S4 Fig — (A) Stability results from the original formulation of the A branch model which includes an initial 2:1 shear stress difference at the flow-convergent bifurcation. (B) Stability results from a version in the model in which the distal branch was 10× longer than the proximal path, resulting in a 10:1 shear stress difference at the bifurcation. We found a similar saddle shape in stability vs. α over time, although much shallower when compared to the original results indicating that bifurcation occurred more readily with the larger shear stress difference. (TIF) [file pcbi.1007715.s018.tif]

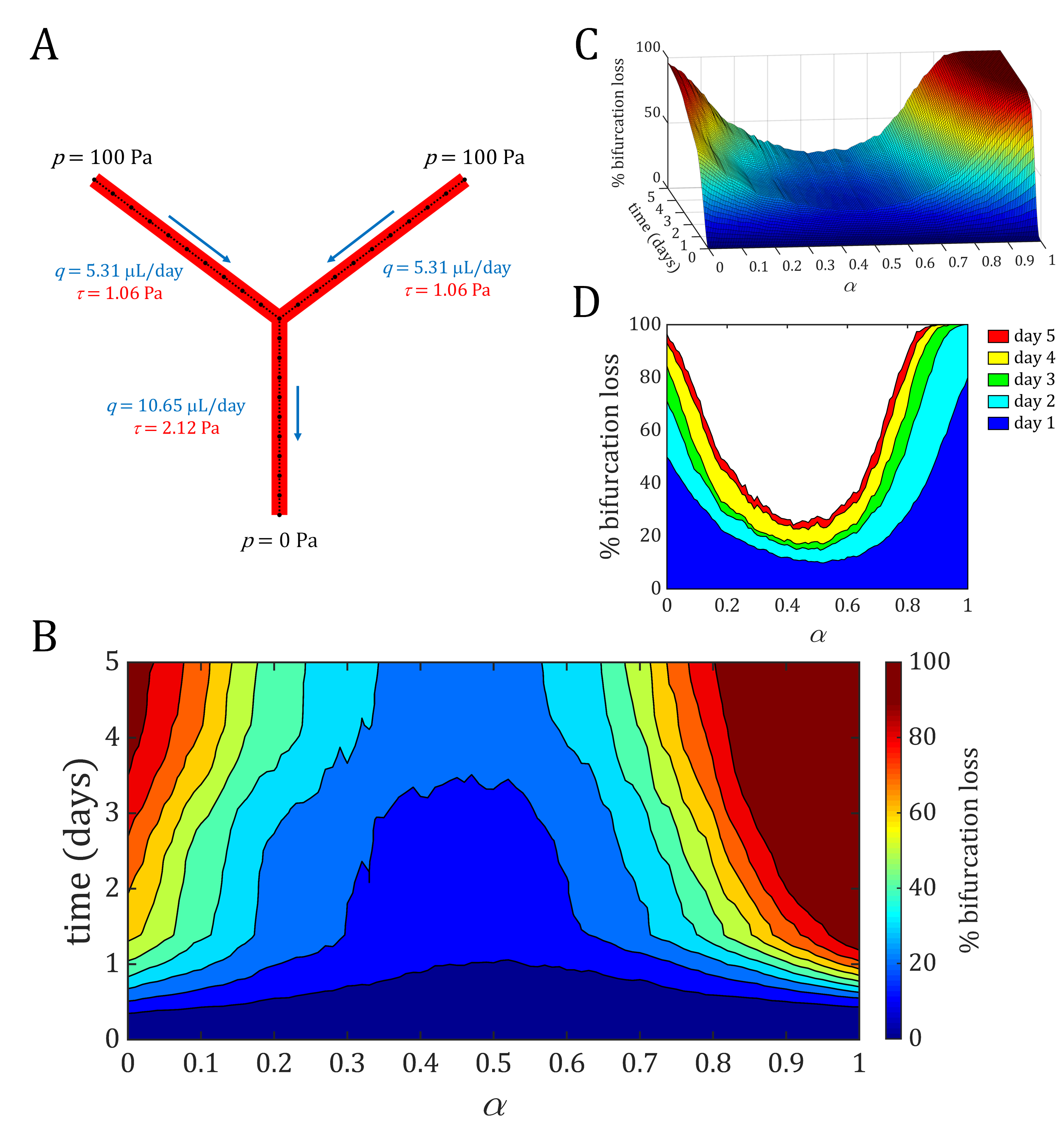

Supplement: S5 Fig — (A) Schematic of the Y branch model with pressure boundary conditions set equal at both inlets, resulting in a 1:1 shear stress at the bifurcation. (B-D) Stability vs. α over time resulted in a similar saddle shape with global minimum centred around α = 0.45 when compared to the A branch model. (TIF) [file pcbi.1007715.s019.tif]

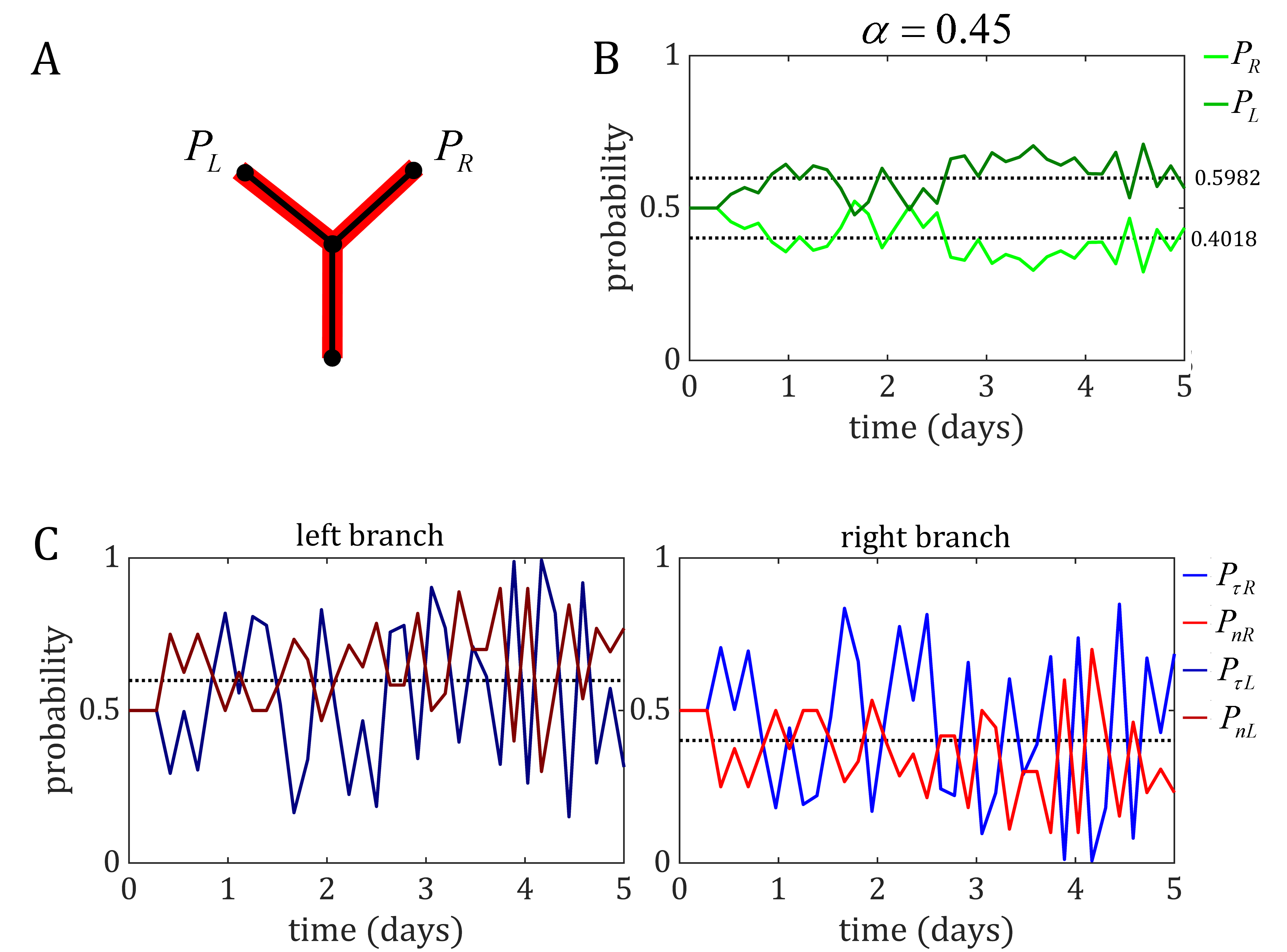

Supplement: S6 Fig — (A) Probability of cells choosing the left branch (PL) or right branch (PR) upon encountering the bifurcation during migration. (B) Probability of each branch initialises at 0.5 (as both branches have the same initial amount of WSS). During the simulation, the branch probabilities tended to separate slightly, favouring one branch over the other. (C) Similar competition between shear stress and cell number probability produces stability at the bifurcation. (TIF) [file pcbi.1007715.s020.tif]

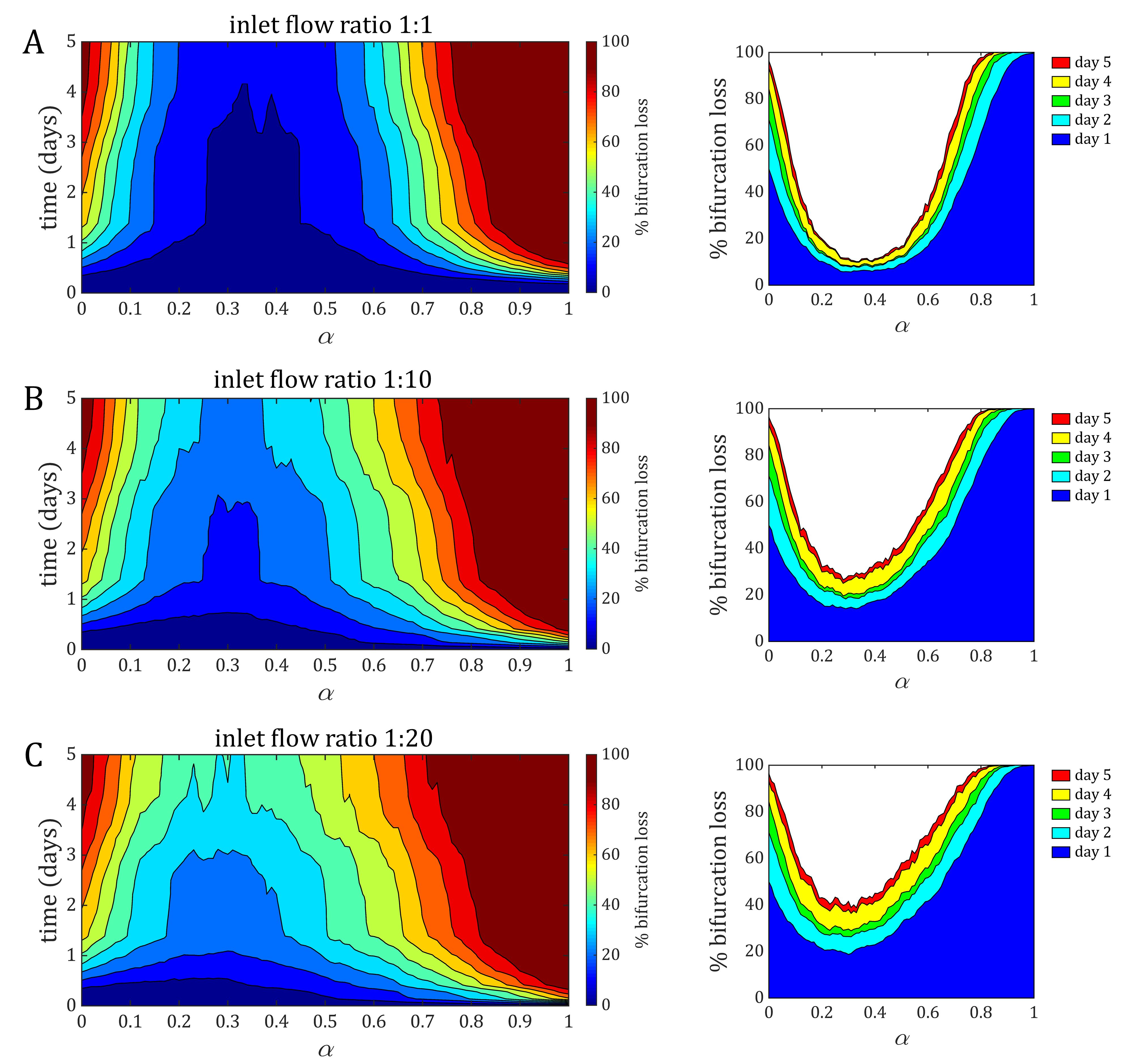

Supplement: S7 Fig — Inlet flow conditions in the Y branch model were used to create initial shear stress ratios between the left and right branch of (A) 1:1, (B) 1:10, (C) 1:20. In general, that stability saddle shifted upward and to the left (towards α = 0.0) as the initial shear stress difference at the bifurcation increased. (TIF) [file pcbi.1007715.s021.tif]

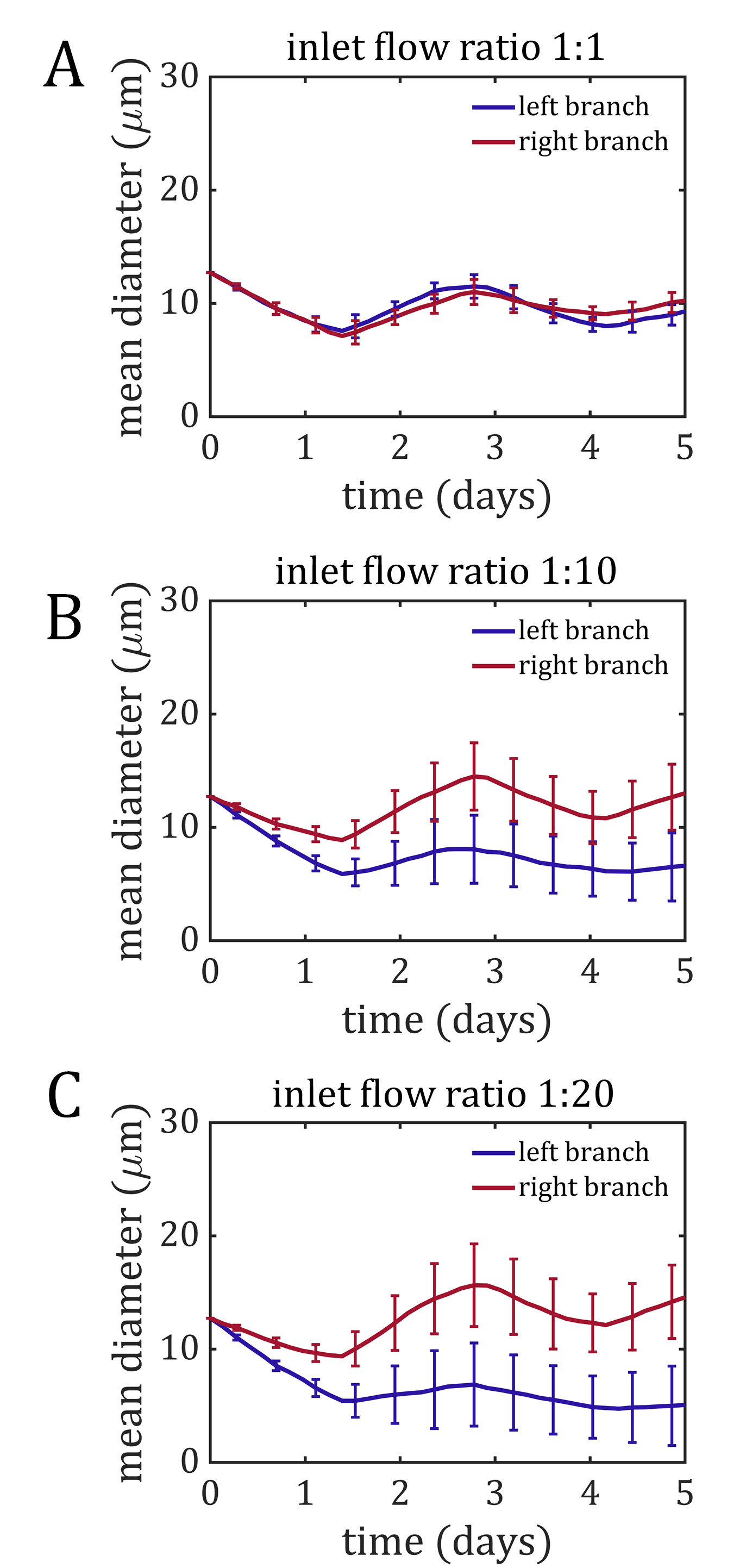

Supplement: S8 Fig — Mean diameter of the left branch (blue) and right branch (red) in the Y branch model with shear stress differences of (A) 1:1, (B) 1:10, and (C) 1:20. The difference in diameter between the two branches increased as this initial shear stress difference increased. (TIF) [file pcbi.1007715.s022.tif]
